# Matricide and schizophrenia- psychopathological, psychodynamic, and forensic aspects: a case report

**DOI:** 10.3389/fpsyt.2023.1240385

**Published:** 2023-08-29

**Authors:** Alexandre Martins Valença, Letícia Rodrigues de Almeida, Gustavo Carvalho de Oliveira, Milena Ferreira França, Antônio Geraldo da Silva, Lisieux Borba Telles, Antonio Egidio Nardi

**Affiliations:** ^1^Department of Psychiatry, Federal University of Rio de Janeiro, Rio de Janeiro, Brazil; ^2^Department of Psychiatry, Fluminense Federal University, Niterói, Brazil; ^3^Department of Psychiatry, University Center of Brasilia, Brasilia, Brazil; ^4^Department of Physician, Escola Superior de Ciências da Saúde, Brasilia, Brazil; ^5^Department of Psychiatry, Universidade de Pernambuco, Recife, Brazil; ^6^Department of Psychiatry, University of Porto, Porto, Portugal; ^7^Department of Psychiatry, Federal University of Rio Grande do Sul, Porto Alegre, Brazil

**Keywords:** violence, homicide, mental disorder, psychosis, matricide, schizophrenia, forensic – psychiatric practice

## Abstract

Matricide is the murder of a mother by her son or daughter, a form of homicide rarely seen in psychiatric practice. A narrative review was conducted on the relationship between matricide and schizophrenia, followed by a case report in Brazil of a schizophrenic patient who murdered his mother and was submitted to forensic psychiatric assessment for criminal liability. The article discusses psychopathological, psychodynamic, and forensic aspects related to the case. The observation of ambivalent and conflictive relations between schizophrenic individuals and their mothers suggests the need for family-level interventions to resolve the understandable occurrence of emotional conflicts, which can serve as stimuli that trigger the murder.

## Introduction

Dostoevsky’s novel *The Brothers Karamazov* (1) tells the story of a troubled family in Russia and the crime of parricide, in which the motive of sexual rivalry is openly admitted. The novel’s hero is Dimitri, but it is his brother who murders their father.

In *Totem and Taboo* (1913) (2), Freud proposed the myth of origin for all humanity and posited parricide as the primeval crime, the foundation of culture. In 1928, Freud’s essay on Dostoevsky (3) resumes this theoretical development initiated in 1913 (2) and reaffirms that parricide is the principal source of guilt feeling. This new approach, which he introduced in *Totem and Taboo* (2) and reaffirmed in “Dostoevsky and Parricide” (3), probably drew on Dostoevsky’s novel as one of its sources of inspiration. Freud viewed *Totem and Taboo* (2) as his most important work. Thus, there could be no better paradigm for such an important scientific breakthrough than one of the world’s greatest novels.

Matricide is defined as the murder of a mother by her son or daughter and is one of the most uncommon forms of homicide, accounting for only 1% to 4% of such crimes (4, 5). Although it is difficult to establish a relationship between mental disorders and specific forms of homicide, various studies suggest that the matricidal aggressor may present schizophrenia or other psychotic disorders (6).

In a systematic review that included 16 studies, Feola et al. (7) reported a total of 80 victims killed by 81 perpetrators (two brothers had committed the matricide in one case). Some 82% of the perpetrators were young males. The most frequent psychiatric diagnosis in the perpetrators was schizophrenia and other psychotic disorders (43%). Sharp force injuries were the leading case of death (55%), followed by blunt trauma (15%) and asphyxia (15%). 14% of the individuals were found not guilty on grounds of insanity, while 26% had diminished criminal responsibility.

A descriptive study on matricide by Green (4) with a sample of 58 male patients admitted to hospital in England found that 74% presented a diagnosis of schizophrenia, 15.5% had a diagnosis of psychotic or endogenous depression, and 10.5% had a diagnosis of personality disorder. The study also found that psychotic symptoms were present in 70% of the cases in the week prior to the matricide. Of those that presented psychotic disorders, 83% were not receiving any kind of treatment at the time of the offense.

A retrospective study of matricides from 1957 to 1987 was conducted in Scotland (5). The study covered cases referred for hospitalization that were considered not criminally liable on grounds of mental insanity, while the incarcerated individuals had been found guilty. A total of 26 cases of matricide were identified, 23 committed by males and three by females. As for psychiatric diagnosis, 10 offenders presented psychotic disorders at the time of the offense (six with a diagnosis of schizophrenia, three of depression, and one of mania), four with alcohol dependence syndrome, and five with personality disorders. Seven did not present any diagnosis. An important finding was that 12 offenders (46%) were intoxicated with alcohol at the time of the offense, including one psychotic individual.

Another retrospective study (8) identified 11 cases of matricide over a 20-year period (1985–2004) in Australia. Ten of the perpetrators were sons and one was a daughter. In all the cases, weapons such as blunt objects (*n* = 5), knives (*n* = 5), firearms (*n* = 3), or ligatures (*n* = 1) were used during the assault. In this sample, mental disorders were present in nine cases (82%), including three with schizophrenia and two with depression. Four other cases were found not guilty of murder on grounds of mental insanity, but without specification of the psychiatric diagnosis.

Bourget et al. (9), in a Canadian study on parricides over the course of 15 years (1990 to 2005), based on consultation of psychiatric records and police files and inquiries into suspicious deaths, identified 64 cases, 37 (57.8%) of which were patricides and 27 (42.1%) matricides. For both offenses, the most frequent mental disorders were schizophrenia and other psychotic disorders (54.2% of matricides and 46% of patricides), followed by depression (16.7% of matricides and 13.9% of patricides) and intoxication with psychoactive substances (4.2% of matricides and 5.6% of patricides). Only four perpetrators (6.3%) were females, three of whom committed matricide. Of these, two presented psychotic disorders and one presented intoxication with a psychoactive substance.

The current study aimed to report a case of matricide committed by a patient with a diagnosis of schizophrenia and referred for forensic psychiatric examination, and to establish psychopathological, psychodynamic, and forensic correlations pertaining to the case. The patient signed a consent form agreeing with the publication of his case.

### Case report

The patient was a 38-year-old single male born in Rio de Janeiro, Brazil, with complete secondary schooling.

The patient was accused of stabbing his mother to death with a knife.

The patient had a history of psychiatric treatment, with three previous admissions to psychiatric clinics. He stated that the reason for his hospitalization was the fact that he had harmed his mother and broken various household objects, including the radio and television set. He also reported that approximately one month before committing the murder, he had stopped taking his antipsychotic medication since he had been feeling well and did not want to spend money on medicines.

The patient’s father had died in a car accident during his childhood. He stated that he had been involved in frequent misunderstandings with this mother, alleging that she was constantly giving him orders. He had already harmed her on previous occasions and reported having attempted to stab her with a knife when he was 15 years old.

The patient had never had girlfriends or female companions. He had engaged previously in homosexual relations. He had no children and was living with his mother, sister, and niece. He was retired, due to mental problems.

Upon psychiatric examination, the patient displayed regular attire and personal hygiene. His speech was poor, showing difficulty in expressing himself and an attitude of strangeness and bewilderment. He showed little modulation of his facial expression over the course of his verbal report. He was lucid, generally oriented, and his memories of recent and past events were preserved.

When asked to talk about the crime’s motivation, he said, “I had problems with my mother. She wanted me to take medication, and one day I decided to take her life… I freaked out, I lost it… I thought that she did not give me the support she could, she showed no understanding… there was a kind of pressure from the TV, I think I’m stalked by people… an indirect pressure, there’s an expectation on me… my mother left me very exposed to those people on the other side… I did not like her watching TV, but she turned the set on all the time… I thought she had dealings with the people on the other side. She had offended my manhood. The people on TV and the songs by the singers were monitoring my life and my thinking, like they wanted to capture what I think… my mother left me more exposed, she collaborated with them, for me to feel insecure and pass this over to them in the communications media.” The patient further reported that his mother criticized him harshly for harboring these beliefs.

The patient’s thinking presented looseness in the associative connections (disaggregation), with impoverished affect and frankly jeopardized volition and pragmatism. He was not displaying hallucinations at the time of the examination. His intelligence was within normal limits and his mood was apathetic. The patient expressed persecutory delusional and self-referential ideation, besides disturbance in self-awareness.

The final psychiatric diagnosis was established through the psychiatric interview and observation of the forensic and hospital records, using DSM-IV-TR criteria (10). The patient in question met the diagnostic criteria for paranoid schizophrenia.

## Discussion

The conclusion in this case was that the patient presented a mental illness in the form of schizophrenia. The diagnosis of schizophrenia was done because the patient presented severe recurrent psychotic symptoms: delusions, formal alterations in thinking, disturbance of self-awareness, ideational and affective impoverishment. In addition, the severity of the mental disorder (schizophrenia) had led to the patient’s previous psychiatric hospitalizations due to his agitation and psychotic symptoms.

The patient’s schizophrenia resulted in his precarious judgement and volition, with a clear causal nexus between the schizophrenic psychosis and the crime in question. The patient’s severe mental illness left him unable to discern the consequences of his acts or to control his aggressive impulses. He was considered not guilty by reason of insanity.

The patient’s actions were noticeably motivated by the presence of his psychotic symptoms, with a clear causal nexus between the symptoms and the crime. In fact, the bpatient’s mother (the victim) entered his delusional system, in which he believed falsely and pathologically that when she turned the television on, she was exposing his life, hence his hostility and aggressiveness towards her.

The forensic psychiatric examination in this case concluded that at the time the crime was committed, the patient was utterly incapable of comprehending and deciding on his acts and was thus not criminally liable (not guilty by reason off insanity) and was referred for psychiatric treatment.

The criterion adopted by Brazil’s penal code (11) to assess criminal liability is biopsychological: such responsibility is only ruled out if the individual, due to mental illness or intellectual disability, is deemed incapable of ethical and legal understanding and self-determination at the time of the act. The biopsychological method requires verification of the actual existence of a causal nexus between the anomalous mental state and the crime committed, that is, that this state, contemporaneous with the act, has partially or completely deprived the individual of any of the above-mentioned psychological capacities (whether intellectual or volitional).

The current case bears several resemblances to other case studies (9, 12): the patient was unemployed, dependent on his mother, and with low socioeconomic status. Various phenomenological risk factors for violent behavior (13) were also present in this case, such as fear and loss of self-control associated with paranoid delusions, including the belief that people on television and the radio were capturing his thoughts and exposing him, facilitated by his mother, in addition to impulsiveness and negative affect (rage towards the mother), all of which contributed to the murder. The mother–child relationship present in the case may justify the hypothesis of the presence of a pathological relationship with the prevalence of an “insecure/ambivalent attachment type” (14).

In cases of matricide it is common the existence of relationship difficulties with the opposite sex, and only immature attempts were made, to separate from the mother and build an independent affective relationship, as in the case in question. The absence of the father figure may contribute to accentuate the complex mother–child relationship, favoring the creation of an exclusive and restricted bond that the child perceives as suffocating, which will contribute to an aggressive behavior against the mother (15).

Psychoanalytical theories have included suggestions that an Oedipal sexual conflict can contribute to a feeling of guilt and the impulse to possess the mother sexually through matricide, or on the contrary, that a pre-Oedipal attachment to the mother and a relationship of dependency pose a threat to the aggressor’s identity (16). Another view is based on the theory of family systems, according to which the primary cause of matricide is attributable to an abusive and pathological family structure that the perpetrator finds unbearable (17).

According to Palermo (18), psychological factors, such as individual predisposition, impulsivity, sudden explosion of repressed anger, behaviors due to psychotic illnesses, and provocation by or misinterpretation of the victim’s behavior, contribute to intrafamilial violence. He summarized such cases under the heading of the so-called “hostile family theory.” According to this author, matricide stems from a combination of unwelcome dependency on the mother and a frustrated desire to be close to her.

Other recurrent features of the mother, as present in this case, include an intrusive character and a tendency to denigrate the son and, in general, to have an ambivalent relationship with him (12).

The literature features various descriptions of the peculiar nature of the mother–child relationship in matricide (12). The bond is often described as “mutually dependent and hostile,” simultaneously conflictive and indissoluble, marked by the child’s difficulty in breaking with the mother’s domination (19). The current study illustrates a conflictive relationship between mother and son.

The patient also felt harshly criticized by his mother, according to his report. In such cases, the mother is often described as a domineering figure in the family, while the father is generally absent (deceased in this case). Another common trait in the mother is the tendency to maintain control over the son, engaging in a sort of “power struggle” (20) based on humiliation to ward off his requests for freedom. These women adopt a paradoxical behavior, characterized by both excessive concern over the consequences of the child’s behavior and harsh criticisms of the child’s incapacity to grow and become an independent adult.

According to the patient’s sister, it was common the existence of disagreements between him and his mother, because she always criticized him for not working and not having a girlfriend, which made him very angry. Still in agreement with the patient’s sister, before the crime the patient had been very angry, having made verbal threats against the mother. It is important to note that he had already assaulted her previously. Certainly, these signs should have motivated the immediate search for the treatment team and the use of antipsychotic medication.

Maybe family interventions could better explore the dynamics of the mother–child relationship, understand the resentments between them, attenuate the mother’s critical behavior towards the son and highlight the importance of drug treatment for the case. Certainly, it would also be important to include the patient’s sister in the family intervention, in order to better understand the functioning of the family dynamics and the sister’s role in family conflicts and her relationship with the patient.

Various studies have found a consistent pattern of interrupted contact with mental health services, while in others, the homicide appears to occur soon after the mental disorder’s onset and before the offender has established contact with these services (21). The patient in question was not in psychiatric or psychotherapeutic treatment or in use of psychiatric medication before the crime. It is important for mental health services to work to prevent loss of contact and lack of treatment adherence, which frequently precede homicides committed by persons with severe mental disorders. It is also crucial for society and government authorities to decrease the barriers to psychiatric and psychosocial treatment.

## Conclusion

The observation that there are ambivalent and conflictive relations between individuals with schizophrenia and their mothers suggests the need for family-level interventions to resolve the understandable occurrence of emotional conflicts, which can act as stimuli that end up triggering the murder (14). It is certainly important for psychiatrists and other mental health professionals to be aware of the risk of violent behavior in patients with a long history of mental illness, with episodes of violence during the acute phase, threats against family members or friends, and lack of regular psychiatric treatment. This professional awareness can thereby contribute to the prevention of such behaviors.

## Data availability statement

The original contributions presented in the study are included in the article/supplementary material, further inquiries can be directed to the corresponding author.

## Ethics statement

Written informed consent was obtained from the individual(s) for the publication of any potentially identifiable images or data included in this article. Written informed consent for the publication of any potential identifying data or images were obtained from the participant. Written informed consent was obtained from the participant/patient(s) for the publication of this case report.

## Author contributions

LA, MF, AS, GO, and LT contributed to conception and design of the study, analyzed the case, and reviewed literature. AN reviewed and organized the sections of the manuscript and rewriting parts of the manuscript. AV wrote the first draft of the manuscript and support review of publication. All authors contributed to the article and approved the submitted version.

## Conflict of interest

The authors declare that the research was conducted in the absence of any commercial or financial relationships that could be construed as a potential conflict of interest.

## Publisher’s note

All claims expressed in this article are solely those of the authors and do not necessarily represent those of their affiliated organizations, or those of the publisher, the editors and the reviewers. Any product that may be evaluated in this article, or claim that may be made by its manufacturer, is not guaranteed or endorsed by the publisher.
